# Music training is associated with cortical synchronization reflected in EEG coherence during verbal memory encoding

**DOI:** 10.1371/journal.pone.0174906

**Published:** 2017-03-30

**Authors:** Mei-chun Cheung, Agnes S. Chan, Ying Liu, Derry Law, Christina W. Y. Wong

**Affiliations:** 1 Department of Social Work, The Chinese University of Hong Kong, Shatin, New Territories, Hong Kong SAR; 2 Department of Psychology, The Chinese University of Hong Kong, Shatin, New Territories, Hong Kong SAR; 3 Chanwuyi Research Center for Neuropsychological Well-being, The Chinese University of Hong Kong, Shatin, Hong Kong SAR; 4 School of Public Administration, Guangzhou University, Guangzhou, P.R. China; 5 Institute of Textiles and Clothing, The Hong Kong Polytechnic University, Hung Hom, Kowloon, Hong Kong SAR; Universitat Zurich, SWITZERLAND

## Abstract

Music training can improve cognitive functions. Previous studies have shown that children and adults with music training demonstrate better verbal learning and memory performance than those without such training. Although prior studies have shown an association between music training and changes in the structural and functional organization of the brain, there is no concrete evidence of the underlying neural correlates of the verbal memory encoding phase involved in such enhanced memory performance. Therefore, we carried out an electroencephalography (EEG) study to investigate how music training was associated with brain activity during the verbal memory encoding phase. Sixty participants were recruited, 30 of whom had received music training for at least one year (the MT group) and 30 of whom had never received music training (the NMT group). The participants in the two groups were matched for age, education, gender distribution, and cognitive capability. Their verbal and visual memory functions were assessed using standardized neuropsychological tests and EEG was used to record their brain activity during the verbal memory encoding phase. Consistent with previous studies, the MT group demonstrated better verbal memory than the NMT group during both the learning and the delayed recall trials in the paper-and-pencil tests. The MT group also exhibited greater learning capacity during the learning trials. Compared with the NMT group, the MT group showed an increase in long-range left and right intrahemispheric EEG coherence in the theta frequency band during the verbal memory encoding phase. In addition, their event-related left intrahemispheric theta coherence was positively associated with subsequent verbal memory performance as measured by discrimination scores. These results suggest that music training may modulate the cortical synchronization of the neural networks involved in verbal memory formation.

## Introduction

Functional [1–14] and structural [14–19] neuroimaging studies investigating the link between music training and brain plasticity have suggested that music training are associated functional and structural modifications in the human brain and changes in cognitive functions. For example, using functional magnetic resonance imaging (fMRI), Pallesen et al. [1] found greater blood oxygenation-level dependent (BOLD) activation in the right lateral prefrontal cortex, lateral parietal cortex, insula, and putamen of musicians during working memory tasks with musical sounds, compared with non-musicians. These regions form neuronal networks that mediate sustained attention and cognitive control. In contrast to those without music training, individuals with music training showed stronger neuronal activation in the hippocampus, frontal and temporal cortices, and the somatosensory areas during an auditory, motor, and somatosensory task [2–4]. Groussard et al. [5] also reported higher neuronal activation in the hippocampus, medial frontal gyrus, and superior temporal gyrus in both hemispheres of individuals with music training during a musical familiarity task that assessed semantic memory for music. In several studies using magnetoencephalography (MEG) to understand the influence of music training on cognitive functioning, individuals with music training showed various advantages, including short-term auditory learning [6–8] and auditory memory capacity for complex auditory patterns [9]. Other MEG studies have also supported the notion that musicians have enhanced auditory and audiovisual processing associated with increased activity in the frontal and temporal regions [10] and in the auditory cortex [11, 12], respectively. Structurally, Groussard et al. [5] revealed greater gray matter density in the hippocampus, an area important for memory processing, in individuals with music training than in those without. These functional and structural brain alterations are associated with the intensity and duration of music practice, that is, the more music practice, the stronger the neuronal changes [20]. More importantly, music training may have a differential effect on brain plasticity and cognitive development depending on the age of commencement [14, 19–21].

Behavior studies suggest that music training is associated with better intellectual functioning [22, 23], speech and language abilities [24], nonverbal reasoning [25], working memory [26–29], verbal memory [26, 30–33], executive functioning [22, 34], and attention span [35, 36]. Among the cognitive functions, the association between music training and memory functioning has been extensively explored using behavioral and neuroimaging techniques over the past two decades [5, 26, 30–33, 37, 38]. College students who received music training before the age of 12 demonstrated better verbal memory than those who did not, suggesting that childhood music training has long-term positive effects on verbal memory [30]. Better verbal memory performance among individuals with music training compared with those without has been replicated in other studies [26, 31–33]. Furthermore, there is a causal relationship between the duration of music training and improvement in verbal memory. After controlling for the effects of age and educational level, a longer duration of music training was significantly associated with better verbal memory in a longitudinal study with a group of children with one to five years of music training. Although some of the children ended their music training after three months of the baseline measurement, their verbal memory performance remained stable at the one-year follow-up, suggesting that the beneficial effects of music training on verbal memory prior to discontinuing the training may be sustained over time [31]. Brandler and Rammsayer [38] compared different aspects of primary mental abilities between individuals with and without music training, and found that those with at least 14 years of music training showed significantly better verbal memory and reasoning. However, Helmbold et al. [39] could not replicate these findings. Some studies have further suggested that the observed enhancements in verbal memory may be transfer effects promoted by music training [20, 40, 41] and that enhanced use of verbal rehearsal [26] and temporal-order processing [42] contribute to better verbal memory in individuals with music training. Therefore, the literature provides evidence of the long-term benefits of music training on cognitive functions, and music training at an early age can systematically shape the development of cognitive functioning.

Memory processing involves the integrated involvement of different brain areas [43]. With the help of the EEG technique, it is possible to examine the cortical synchronization patterns underlying memory processing, thereby giving information about the functional dependencies of the respective brain areas [44]. Coherence in the theta frequency band is highly associated with memory processing. Specifically, greater long-range coherence in the theta frequency band between the anterior and posterior brain regions is associated with increased working memory demands [45–47] and the successful encoding and storage of episodic information [48–52]. In particular, Summerfield and Mangels [49] found that successful item encoding was characterized by elevated long-range left intrahemispheric coherence in the theta frequency band. Theta oscillation in the right hemisphere only occurred when processing additional contextual information (e.g. word-color associations). Higher short-range intrahemispheric theta coherence and interhemispheric theta coherence between anterior and posterior cortical regions were also associated with the later recall of concrete and abstract nouns, respectively [50]. During memory tasks involving manipulation rather than simple retrieval and maintenance of information, there was an increase in theta coherence between the frontal and parietal cortical regions [44]. Although previous research found that verbal learning with a musical template was associated with increased intra- and interhemispheric EEG coherence in the theta frequency band [37], it remains unknown whether individuals with music training really manifest an increase in their long-range theta coherence during the verbal memory encoding phase that can be attributed to superior memory performance as a result of music training. Thus, the purpose of this study was first to examine the association between music training and memory performance in both visual and verbal modalities, and second to investigate the neurophysiological correlates underlying the verbal memory encoding of individuals with and without music training as indicated by their EEG coherence patterns.

Given the evidence from existing empirical studies of the potential benefits of music training for verbal episodic memory, we anticipate that individuals with music training demonstrate better learning and recall of learned verbal material than those without music training. According to the selective benefits of music training on verbal memory reported in previous studies [26, 30–33, 37, 38], we predict better learning of verbal but not visual material in individuals with music training, and we therefore use a visual memory test as the control task in this study. In view of increased long-range theta coherence in relation to better memory recall [48–52], those with music training demonstrate altered, probably enhanced, long-range theta coherence during the verbal memory encoding phase, compared with individuals without music training. This alteration is also anticipated to be more prominent in left intrahemispheric coherence, but with possible changes in right intrahemispheric and interhemispheric coherence, which are hypothesized upon the reported association between left intrahemispheric theta coherence and successful encoding and recall of verbal materials [49] and the increased intra- and interhemispheric theta coherence during verbal learning with a musical template [37]. Given the previous findings of elevated short-range coherence during memory processing [37, 50], higher short-range theta coherence is expected among individuals with music training, particularly over the frontal scalp region. As we further anticipate the distinct neurophysiological pattern in individuals with music training to be correlated with their memory performance during the EEG paradigm, we hypothesize that long-range theta coherence during the verbal memory encoding phase is positively associated with verbal memory performance in the EEG experiment. Finally, as the literature suggests the moderating effect of years of music training on neuronal changes [20] and verbal memory [31], we also predict that more years of music training result in better verbal memory performance and greater changes in theta coherence.

## Materials and methods

### Participants

Sixty healthy participants who reported a negative history of neurological and psychiatric problems took part in the study. All of them were right-handed. The study was conducted in accordance with the Helsinki Declaration of the World Medical Association Assembly, and the research protocol was approved by the Human Subjects Ethics Sub-committee of The Hong Kong Polytechnic University. All of the participants took part voluntarily and signed an informed consent form in accordance with institutional guidelines.

Half of the participants (MT, mean age 21.03 ± 1.16) had received formal music training in Chinese (e.g. erhu, pipa) or Western instruments (e.g. piano, violin, or flute) on a regular basis with a minimum of one hour each week in their free time for at least one year. Table 1 presents the types of musical instruments the participants played and their duration of practice.

**Table 1:** Types of musical instruments and duration of practice.

Types of musical instruments	Number of participants	Duration of practice (years)
Western Instruments		
Piano	17	1.6–13
Guitar	3	1–4
Melodica	2	1–9
Recorder	2	2.1–3
Trombone	1	2
Percussion	2	1.2–3
Chinese Instruments		
Erhu	1	4
Pipa	1	7
Zheng	1	9

Besides music lessons at school, the other 30 participants (NMT, mean age 20.93 ± 1.26) had never received formal music training during their free time. The two groups were matched (*p* > 0.05) for age, education, gender, and cognitive capability, as estimated by the Test of Nonverbal Intelligence IV [53] which was individually administered to the participants. Trials were presented with increasing difficulty, and each trial comprised a sequence of abstract figures with one figure missing. Based on attributes such as shape, position, direction, rotation, or shading [53], the participants chose the correct figure from an array of six choices. Correct answers earned one point and the total scores obtained were then converted to an estimated IQ score. The demographic characteristics of the participants are shown in Table 2.

**Table 2:** Demographic characteristics and behavioral measures of verbal memory in EEG paradigm.

	Without music training (NMT, n = 30)	With music training (MT, n = 30)	t / χ2	p - value
Age (years)	20.93 ± 1.258	21.03 ±1.159	-0.320	0.750
Education (years)	15.066 ± 0.254	15.067 ± 0.254	0.000	1.000
Gender (M/F)	3/27	5/25	0.577	0.448
Years of music training	N.A.	5.210 ± 3.864	N.A.	N.A.
Estimated IQ	101.900 ± 14.470	103.800 ± 15.925	-0.484	0.630
Discrimination score (%)	80.139 ± 11.460	87.639 ± 10.351	-2.660	0.010
Reaction time to correct hits (ms)	841.563 ± 132.966	858.701 ± 145.809	-0.476	0.636
Reaction time to false alarms (ms)	939.421 ± 193.954	933.459 ± 197.075	0.118	0.906

N.A. = not applicable

### Neuropsychological assessment of memory functioning

#### Verbal memory

The Hong Kong List Learning Test—Form One (HKLLT) [54], a locally validated verbal-memory test [30, 55–61], was used to assess the verbal memory of the participants. The list learning test, which consists of a list of 16 two-character Chinese words, was presented orally three times to each participant (Trials 1 to 3). They were asked to recall as many words as possible immediately after each learning trial, and then again after 10 (Trial 4) and 30 minutes (Trial 5). The maximum possible score for each of the 5 learning trials was 16 words.

#### Visual memory

The Visual Reproduction subtest (VR) of the Wechsler Memory Scale-III (WMS-III) [62] was used to assess the visual memory of the participants. Five designs with different levels of complexity were presented for 10 seconds each. The participants were then required to recall as many details of each design as possible immediately after its presentation, and again after 30 minutes. The maximum score for the test was 104 points. The visual memory test was used as a control condition, which was anticipated to have a negligible association with music training compared with the verbal memory test.

### EEG recording

The EEGs were recorded in a Faraday chamber with 64 Ag/AgCl-sintered electrodes mounted on a stretch-lycra Quik-Cap (Neuroscan, El Paso, TX, USA). The electrodes were placed in accordance with the international 10–10 system [63]. A ground electrode was placed on the forehead anterior to the Fz electrode. The linked-ears reference scheme was used throughout the data acquisition. Vertical electrooculograms (VEOGs) were recorded between the supra- and sub-orbit electrodes of the left eye, whereas horizontal electrooculograms (HEOGs) were recorded between the left and right outer canthus electrodes. The electrode impedance was less than 10 kΩ and homologous sites were within 1 kΩ of each other. Quik-Gel (El Paso, TX, USA) was used as the conducting medium. The signals were amplified with a Neuroscan SynAmps^2^ amplifier unit (El Paso, TX, USA) with a band-pass filter of 0.05–200 Hz and digitized at a sampling rate of 1000 Hz.

### EEG paradigm

**Fig 1:**
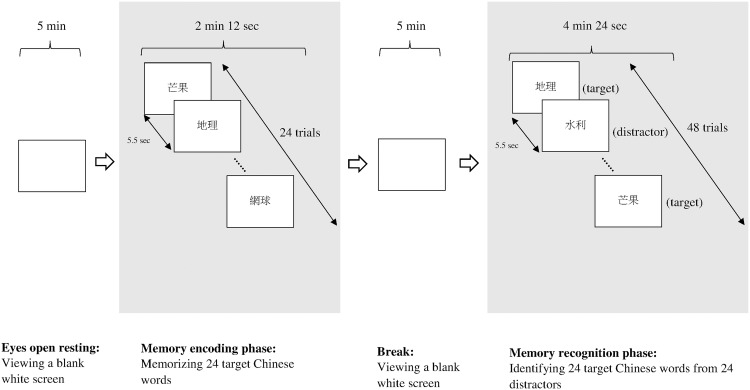
The memory paradigm during EEG recording.

The EEG data were recorded under two conditions in the following sequence: 1) resting with eyes open as the baseline condition, and 2) during the verbal memory encoding as the experimental condition (Fig 1). During resting with eyes open, the participants were asked to fully relax and look at the blank white screens of their respective computer monitors for five minutes. The EEG data for this condition were regarded as the baseline for their EEG activity. During the verbal memory encoding phase, the participants were asked to memorize a list of 24 distinct two-character Chinese words [55, 56], which were individually presented for 5 seconds each with an inter-stimulus interval of 5.5 seconds. The duration of the verbal memory encoding phase was approximately two minutes. The two-character Chinese words, which were completely different from those used in the HKLLT, were selected from four categories of a set of normative data for bilingual students in Taiwan [64]. Half were concrete words from categories of fruit and instruments, and the other half were abstract words from categories of occupations and sport. The stimuli were generated and controlled by the stimulus presentation software, the Neuroscan Stim^2^ Complete System (El Paso, Texas, USA), and presented to the participants in the center of a 19-inch color CRT monitor. To avoid interference between the list learning trials used in the neuropsychological assessment and during EEG recording, the participants had to attend sessions scheduled on two different dates and were allowed to choose their preferred first session. The average time lag between the neuropsychological assessment and the EEG recording session was around 16.02 days (SD 13.33 days).

### EEG data processing

The EEG data for the two conditions were all collected with the Neuroscan system and then processed offline using the NeuroGuide software program (NeuroGuide, v.2.5.2, http://www.appliedneuroscience.com). Individual EEG files were first visually inspected by a trained technician who was blind to the purpose of the study to detect any type of gross abnormalities in the data. An artifact-free segment of at least 10 seconds was selected and used as the template for detection of eye movement and blink artifacts and drowsiness in the EEG data. Using this selected artifact-free EEG template, the data were algorithm edited by the built-in automated artifact detection and rejection toolbox in the software to remove all non-EEG artifacts. The sensitivity level of the automated artifact rejection tool was set at the default threshold of 1.5 standard deviations from the amplitude of the artifact-free EEG template, such that a segment was rejected if it contained at least 1 second of successive instantaneous z scores above 1.5 standard deviations. The edited segments were then statistically analyzed for split-half and test-retest reliability to compute the reliability coefficient for each electrode site. Split-half reliability was defined by the ratio of the variance between even and odd seconds of the edited selections, whereas test-retest reliability was defined by the ratio of the variance between the first and second half of the selection. Zero phase-shift band pass filtering between 0.5 and 30 Hz was also applied to each of the artifact-free EEG data segments. Only segments with at least one minute of artifact-free data showing minimum split-half and test-retest reliability ratios of 0.90 were subsequently entered into the NeuroGuide’s spectral analysis system for fast Fourier transformation (FFT). In the FFT, all of the selected artifact-free EEG data were spliced together as a series of 256 digital values with an epoch length of 2 seconds using a sampling rate of 128 samples per second and a cosine taper window. To minimize the effects of windowing in the FFT, the EEG segments were processed with a sliding average of 75% overlap. Based on this, the raw EEG data from each channel were divided into predefined frequency ranges by the software. In this study, the EEG data were analyzed over 64 electrode positions in the theta (4–8 Hz) frequency band only, as the theta rhythm is important for memory encoding [43, 45, 48–52, 65]. The long-term potentiation that forms enduring memory traces appears to be induced or at least enhanced through stimulation in the theta frequency band [66, 67]. Secondly, the hippocampus, an important brain region that mediates memory formation, is found to be one of the source generators of the theta frequency band [68, 69]. The linkage between the hippocampal theta rhythm and memory formation has also been validated in human studies [70–73].

Theta coherence, defined as the spectral cross-correlation between two signals normalized by their power spectra [74, 75], was calculated between all possible pairings of the 64 electrode sites except for the eight midline electrodes (Fpz, Fz, FCz, Cz, CPz, Pz, POz, and Oz). Fisher’s *z*-transform was used to produce a Gaussian distribution from coherence values. Following the published literature [76–78], the values obtained were inverse-transformed for reporting. They ranged from 0 to 1, with higher values representing stronger phase synchronization between the signals of two electrode pairs. Short-range intrahemispheric coherence denotes the synchronization of brain signals measured at adjacent electrode pairs, and is a measure of functional connectivity within the local scalp regions. Long-range coherence describes the synchronization of brain signals measured at any electrode pair that is separated by at least one electrode, and reflects the functional connectivity between two distal scalp regions of the hemispheres.

Theta coherence values for possible electrode pairs were further averaged and categorized as follows: (i) short-range intrahemispheric coherence (between adjacent electrode pairs, F1–F3, C3–C5, P5–P7 (left) versus F2–F4, C4–C6, P6–P8 (right)); or (ii) long-range intrahemispheric coherence (separated by at least one electrode, F1–C1, C3–P3, F5–P5 (left) versus F2–C2, C4–P4, F6–P6 (right)). Interhemispheric coherence was separately measured at the electrode sites over the following scalp regions: (i) frontal (Fp1–Fp2, AF3–AF4, F1–F2, F3–F4, F5–F6, F7–F8), (ii) central (FC1–FC2, FC3–FC4, FC5–FC6, C1–C2, C3–C4, C5–C6, CP1–CP2, CP3–CP4, CP5–CP6), (iii) temporal (FT7–FT8, T3–T4, TP7–TP8), and (iv) parietal/occipital (P1–P2, P3–P4, P5–P6, P7–P8, PO3–PO4, PO5–PO6, PO7–PO8, O1–O2) [79–81].

### Behavioral measures of verbal memory in the EEG paradigm

After the participants finished the EEG task, they were allowed a 5-minute break during which they were asked to fully relax and look at the blank white screen of their respective computer monitor (Fig 1). Then they were presented with a recognition trial to discriminate between the 24 two-character Chinese words memorized during the verbal memory encoding phase and another 24 two-character Chinese words used as distractors. Each Chinese word was presented on the computer screen for 5 seconds and the participants were asked to discriminate between them by pressing buttons on an STIM Response Pad. The discrimination scores during recognition using the formula [(correct hits—false alarms)/24 *100%], which ranged from -100% to 100%, were calculated and regarded as an index to reflect verbal memory performance in the EEG experiment. Discrimination scores were used instead of correct hit scores because the former take false alarms into account and are therefore a better measure of memory accuracy. The reaction times for correct hits and false alarms were also recorded and compared.

### Statistical data analysis

The statistical data analysis was conducted using SPSS Version 21.0 for Windows (SPSS Inc., Chicago IL, USA). Kolmogoroff-Smirnov tests were used to confirm the normal distribution of the data. As all of the variables showed a normal distribution, parametric statistics, such as independent-samples *t*-test and chi-square test, were used for between-group comparisons, whereas bivariate correlations and linear regressions were performed to test for associations. Repeated-measures analysis of variance (ANOVA) was used to explore the following differences between the groups: verbal and visual memory performance in the neuropsychological assessment, short-range and long-range intrahemispheric theta coherence, and interhemispheric theta coherence. Partial eta-squared (partial η^2^) values were calculated as effect size indices for all of the ANOVA tests. For significant main factors and interactions resulting from the ANOVAs, further post-hoc comparisons were made using independent or paired *t*-tests. A significance level of *p* < 0.005 was used as a Bonferroni correction for multiple comparisons, but results with *p* < 0.01 were also reported to show the trend in the differences.

## Results

### Neuropsychological assessment of memory functioning

#### Verbal memory

**Fig 2:**
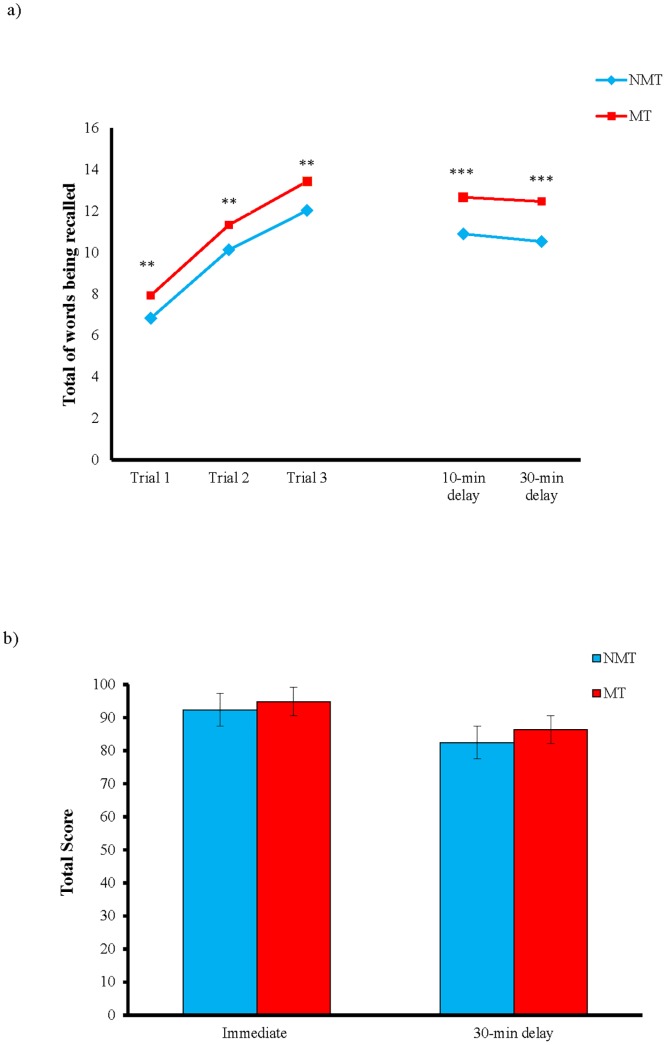
(a) Mean number of words recalled in learning trials 1–3, 10-minute and 30-minute delayed recall trials for the NMT and MT groups on the Hong Kong List Learning Test and (b) mean score of figures recalled in the immediate and 30-minute delayed recall trials on the Visual Reproduction subtest of the Wechsler Memory Scale—Third edition. ** *p* < 0.01 (uncorrected), *** *p* < 0.001 (Bonferroni corrected).

The numbers of words recalled by the participants in the three learning trials are shown on the left side of Fig 2(a), and those recalled in the two delayed recall trials are presented on the right side. A Group (MT, NMT) x Learning Trial (Trials 1 to 3) repeated measures ANOVA was conducted to investigate the possible effect of music training on verbal learning ability. The multivariate results showed a significant main effect of Group, *F*(1, 58) = 14.995, *p* < 0.001, partial, η^2^ = 0.205. Post-hoc pairwise comparison suggested that the participants in the MT group generally recalled more words than did those in the NMT group across the three learning trials (*p* < 0.001). The main effect of Learning Trial, *F*(2, 57) = 241.516, *p* < 0.001, partial, η^2^ = 0.894, was also significant. Post-hoc paired *t*-test showed that the participants generally learned more words in the second trial than in the first, *t*(59) = 14.550, *p* < 0.001, and more in the third trial than in the second, *t*(59) = 8.588, *p* < 0.001. The Group x Learning Trial interaction, *F*(2, 57) = 0.192, *p* > 0.05, was not significant, indicating a similar verbal learning rate in the two groups.

A Group (MT, NMT) x Delayed Recall Trial (Trials 4 & 5) repeated measures ANOVA was used to analyze the number of words recalled in the 10- and 30-minute delayed recall trials to examine the effect of music training on the delayed recall (Fig 2(a)). The main effect of Group, *F*(1, 58) = 20.365, *p* < 0.001, partial η^2^ = 0.260, was significant. Post-hoc independent *t*-test demonstrated that the participants in the MT group generally had better verbal recall than did those in the NMT group for both the 10-, *t*(58) = 4.128, *p* < 0.001 and 30-minute, *t*(58) = 4.552, *p* < 0.001, delayed recall trials, respectively. The main effect of Delayed Recall Trial, *F*(1, 58) = 5.849, *p* = 0.019, partial η^2^ = 0.092, and the Group x Delayed Recall Trial interaction, *F*(1, 58) = 0.506, *p* > 0.05, were not significant after the Bonferroni correction.

While the MT group demonstrated better verbal memory than the NMT group in the delayed recall trials, it was unclear whether their better delayed recall performance was related to a lower rate of forgetting after a delay (i.e. they were able to retain more learned information) or a greater capacity to remember during the learning trial (i.e. a greater learning capacity). Therefore, the rate of forgetting, that is, the amount of information learned at the third learning trial (i.e. Trial 3) that was forgotten after 30 minutes (i.e. Trial 5), was calculated using the formula [(Trial 3–Trial 5)/Trial 3 * 100%], such that the higher the rate, the more information forgotten. Independent-samples *t*-test showed no significant difference in the rate of forgetting between the MT and NMT groups, *t*(58) = 1.826, *p* > 0.05. The comparable rate of forgetting between the two groups suggested that the better delayed recall performance in the MT group was probably not related to a better storage ability than the NMT group. Next, linear regression analysis was performed to examine whether verbal memory in the delayed recall trials was affected by their performance in the learning trails. The results showed that the total number of words remembered across the three learning trials significantly predicted the number of words recalled in the two delayed recall trails in both the MT group (10 minute, *F*(1, 28) = 12.535, *p* = 0.001, *R*^2^ = 31%; 30-minute, *F*(1, 28) = 10.849, *p* = 0.003, *R*^2^ = 28%) and the NMT group (10 minute, *F*(1, 28) = 13.600, *p* = 0.001, *R*^2^ = 33%; 30-minute, *F*(1, 28) = 11.327, *p* = 0.002, *R*^2^ = 29%). These results suggested that the enhanced verbal recall after a delay demonstrated by individuals with music training was due to their greater learning capacity, which allowed them to memorize more words during the three learning trials. Nevertheless, the bivariate correlation analysis showed that total years of music training was not significantly correlated with all learning trials and delayed recall trials [Trial 1: *r*(30) = -0.176, *p* > 0.05; Trial 2: *r*(30) = -162, *p* > 0.05; Trial 3: *r*(30) = 0.045, *p* > 0.05; Trial 4: *r*(30) = -0.191, *p* > 0.05; Trial 5: *r*(30) = -0.182, *p* > 0.05).

#### Visual memory

Fig 2(b) displays the mean scores of the MT and the NMT groups obtained in the two delayed recall trials of the WMS-III VR. A Group (MT, NMT) x Recall Condition (immediate, 30-minute delayed) repeated measures ANOVA was used to analyze the recall scores for the immediate and 30-minute delayed recall trials to examine the effect of music training on visual recall. The multivariate results showed that the main effect of Group, *F*(1, 58) = 1.315, *p* > 0.05, and the Group x Recall Condition interaction, *F*(1, 58) = 0.195, *p* > 0.05, were not significant, suggesting no significant difference in visual memory across recall conditions between the two groups. The main effect of Recall Condition, *F*(1, 58) = 32.317, *p* < 0.001, partial η^2^ = 0.358, was significant. As shown by post-hoc paired-samples *t*-test, the participants performed better in the immediate recall trial (*M =* 93.57, *SD* = 8.44) than in the 30-minute delayed recall trial (*M* = 84.35, *SD =* 15.77), *t*(59) = 5.724, *p* < 0.001. In general, the neuropsychological results were consistent with our previous studies [30, 31], and supported our hypothesis that music training selectively affects verbal but not visual memory.

### Theta coherence during resting with eyes open

A Group (MT and NMT) x Side (left and right) x Range (short and long) repeated measures ANOVA was conducted to compare intrahemispheric coherence in the theta frequency band during resting with eyes open to see if there was any difference between the MT and NMT groups. The multivariate results showed that the main effect for Group, *F*(1, 58) = 2.637, *p* > 0.05, the Group x Range interaction, *F*(3, 56) = 0.014, *p* > 0.05, the Group x Side interaction, *F*(1, 58) = 0.362, *p* > 0.05, and the Group x Side x Range interaction, *F*(1, 58) = 0.725, *p* > 0.05 was not significant. There were significant main effects for Side, *F*(1, 58) = 5078.358, *p* < 0.001, Range, *F*(1, 58) = 35.233, *p* < 0.001, and the Side x Range interaction, *F*(1, 58) = 29.749, *p* > 0.001. Post-hoc pairwise comparison suggested that left intrahemispheric theta coherence (*M* = 0.750) was significantly higher than right intrahemispheric theta coherence (*M* = 0.722), *p* < 0.001, whereas short-range intrahemispheric theta coherence (*M* = 0.926) was significantly higher than long-range intrahemispheric theta coherence (*M* = 0.545), *p* < 0.001. In addition, the difference between long-range and short-range right intrahemispheric theta coherence (*M* difference = 0.395) was larger than the difference between long-range and short-range left intrahemispheric theta coherence (*M* difference = 0.369).

Another repeated measures ANOVA was used to compare the two groups in terms of interhemispheric theta coherence over the four scalp regions (frontal, temporal, central, and parietal/occipital). There was also no significant difference in the main effect of Group, *F*(1, 58) = 2.605, *p* > 0.05 and the Group x Region interaction, *F*(3, 56) = 1.156, *p* > 0.05, whereas the main effect of Region, *F*(3, 56) = 600.430, *p* < 0.001, was significant. Post-hoc pairwise comparison suggested that interhemispheric theta coherence over the four scalp regions was significantly different from each other, with the highest over the parietal/occipital scalp (*M* = 0.724) region, followed by the frontal (*M* = 0.628), temporal (*M* = 0.382) and central (*M* = 0.275) scalp regions (*p* < 0.001). In general, the two groups did not differ significantly in their intrahemispheric and interhemispheric theta coherence at baseline.

### Theta coherence during verbal memory encoding phase

**Fig 3:**
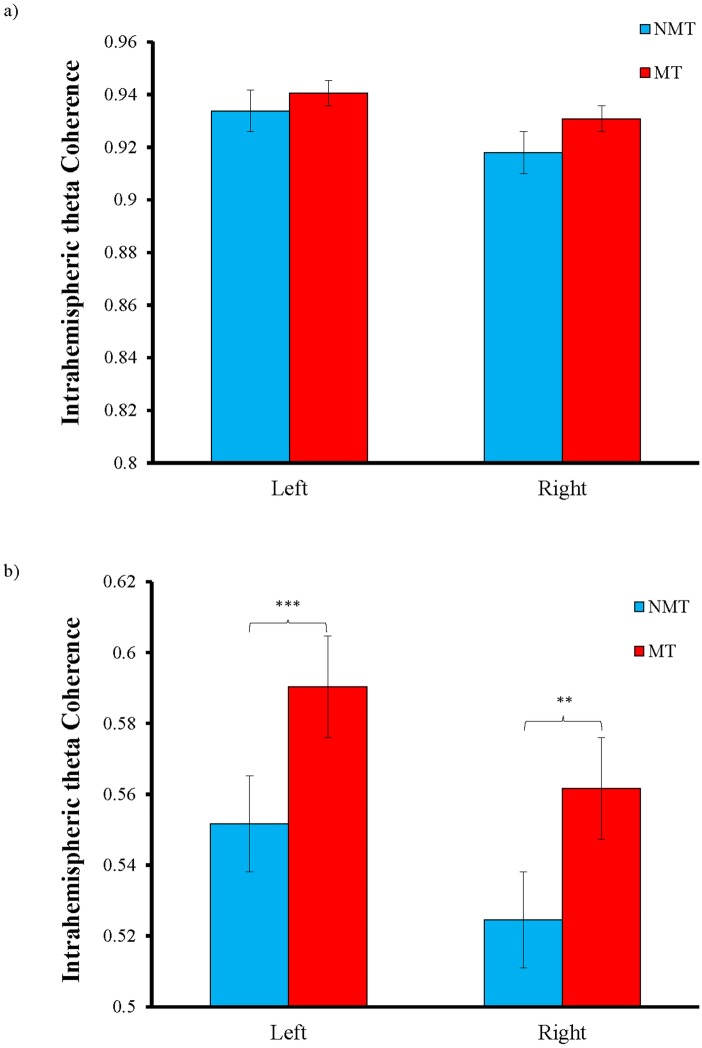
Mean (inverse Fisher’s z) values in the theta (4–8 Hz) frequency band for intrahemispheric (a) short-range coherence and (b) long-range coherence. Compared with the NMT group, long-range left and right intrahemispheric theta coherence is significantly higher in the MT group. ** *p* < 0.01 (uncorrected), *** *p* < 0.001(Bonferroni corrected).

A Group (MT and NMT) x Side (left and right) x Range (short and long) repeated measures ANOVA was conducted to compare intrahemispheric coherence in the theta frequency band for both groups during the task in the EEG paradigm. The results showed that the Group x Range interaction was significant, *F*(1, 58) = 13.989, *p* < 0.001, partial η^2^ = 0.194. Post-hoc pairwise comparison revealed that the MT group had significantly higher long-range intrahemispheric theta coherence than the NMT group during the verbal memory encoding phase (*M* difference = 0.038, *p* < 0.001), but there was no significant difference in short-range intrahemispheric theta coherence (*M* difference = 0.010, *p* > 0.01) (Fig 3). The Group x Side, *F*(1, 58) = 0.070, *p* > 0.05, and the Group x Side x Range interaction, *F*(1,58) = 0.696, *p* > 0.05, were not significant, whereas the main effects of Side, *F*(1, 58) = 22.030, *p* < 0.001, Range, *F*(1, 58) = 9949.993, *p* < 0.001, and the Side x Range interaction, *F*(1, 58) = 10.645, *p* = 0.002, were significant or near the Bonferroni corrected significance level of *p* < 0.005. Post-hoc pairwise comparison suggested that left intrahemispheric theta coherence (*M* = 0.754) was significantly higher than right intrahemispheric theta coherence (*M* = 0.734), *p* < 0.001, whereas short-range intrahemispheric theta coherence (*M* = 0.931) was significantly higher than long-range intrahemispheric theta coherence (*M* = 0.557), *p* < 0.001. In addition, the difference between long-range and short-range right intrahemispheric theta coherence (*M* difference = 0.381) was larger than the difference between long-range and short-range left intrahemispheric theta coherence (*M* difference = 0.366). A bivariate correlation was performed between total years of music training and long-range intrahemispheric theta coherence, but the results showed no significant correlation between the two (left: *r*(30) = 0.035, *p* > 0.05; right: *r*(30) = 0.261, *p* > 0.05).

**Fig 4:**
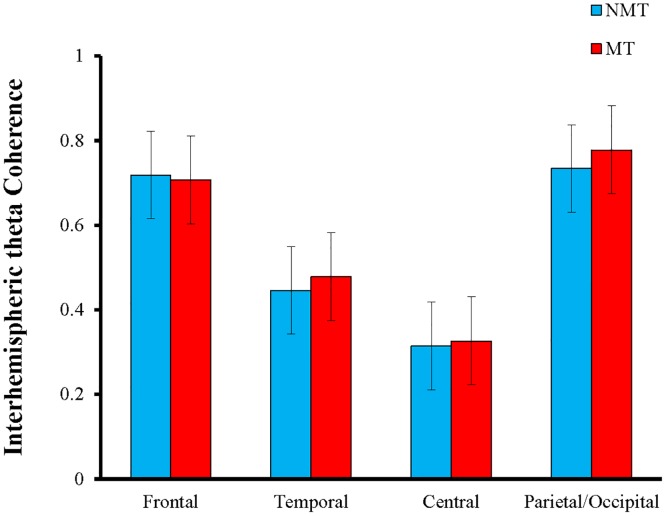
Mean (inverse Fisher’s z) values in the theta (4–8 Hz) frequency band for interhemispheric coherence measured over the frontal, temporal, central, and parietal/occipital scalp regions.

A repeated measures ANOVA was used to compare the two groups in terms of interhemispheric theta coherence over the four scalp regions (frontal, temporal, central, and parietal/occipital). There was no significant difference in the main effect of Group, *F*(1, 58) = 0.859, *p* > 0.05 and the Group x Region interaction, *F*(3, 56) = 2.325, *p* > 0.05 (Fig 4), but the main effect of Region, *F*(3, 56) = 875.668, *p* > 0.001, was significant. Post-hoc pairwise comparison suggested that interhemispheric theta coherence over the frontal (*M* = 0.712) and parietal/occipital scalp (*M* = 0.756) regions was significantly higher than that over the temporal (*M* = 0.461) and central (*M* = 0.320) scalp regions (*p* < 0.001), whereas interhemispheric theta coherence over the temporal scalp region was also significantly higher than that over the central scalp region (*p* < 0.001). In general, the two groups did not differ significantly in their interhemispheric theta coherence during the verbal memory encoding phase.

### Behavioral measures of verbal memory in the EEG paradigm

**Fig 5:**
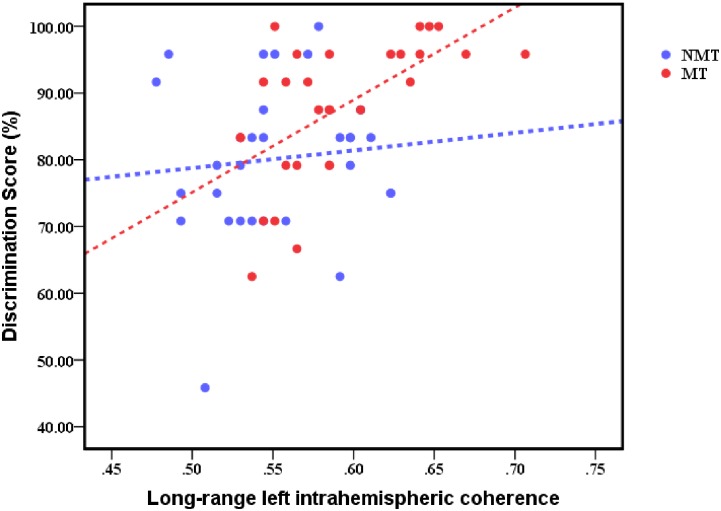
Bivariate correlation between discrimination scores of recognition performance in the NMT and MT groups and their long-range left intrahemispheric coherence in the theta frequency band during the verbal memory encoding phase. (NMT, *r*(30) = 0.097, *p* > 0.05; MT, *r*(30) = 0.562, *p* = 0.001).

An independent samples *t*-test of verbal memory performance as measured by the discrimination scores revealed that the MT group performed better than the NMT group, *t*(58) = -2.660, *p* = 0.01 (Table 2) and the difference was close to the corrected significance level. The bivariate correlation analysis (Fig 5) showed that the discrimination score of recognition performance in the MT group was significantly positively correlated with long-range left intrahemispheric theta coherence during the verbal memory encoding phase in the EEG paradigm, *r*(30) = 0.562, *p* = 0.001. Nevertheless, there was no significant correlation between total years of music training and the discrimination scores, *r*(30) = -0.040, *p* > 0.05. The independent samples *t*-tests showed that the two groups did not differ significantly in the reaction time for correct hits, *t*(58) = -0.476, *p* > 0.05, or false alarms, *t*(58) = 0.118, *p* > 0.01 (Table 2).

## Discussion

Music training is known to be associated with functional changes in the brain. When engaging in cognitive tasks, stronger neuronal activation has been observed in the hippocampus, medial frontal gyrus, temporal cortex, and somatosensory areas of individuals with music training [1–14]. Furthermore, previous EEG studies have revealed that increased long-range theta coherence, particularly between the frontal and posterior scalp regions, is associated with better memory performance [43, 48–52]. This study supplemented the current research on functional changes in the brain associated with music training by comparing EEG coherence in the theta frequency band between individuals with and without music training during the encoding phase of verbal memory. Our findings suggested that the difference in brain activity during the verbal memory encoding phase was associated with enhanced verbal memory recall. Therefore, the neurophysiological changes in individuals with music training during the verbal memory encoding phase agree well with previous studies that suggest an association between music training and brain plasticity.

Consistent with previous behavioral studies [26, 30–33], the participants with music training in this study demonstrated better memory performance than individuals without music training in both learning and delayed recall trials of a verbal list learning test. No significant difference in memory performance was found in the visual memory test. In addition, the two groups demonstrated a similar rate of forgetting. Enhanced verbal recall in the delayed recall trials was significantly associated with better performance during the learning trials, regardless of whether there was music training. Therefore, it seems that music training is associated with the capacity to learn and remember more information during the verbal memory encoding phase, as well as better verbal recall performance after a delay. One possible explanation is that the improved performance is due to better use of rehearsal mechanisms with the verbal material during both the learning and delayed recall trials [26].

In terms of enhanced learning capacity, the cortical synchronization of memory processing between the EEG channels for individuals with and without music training during the verbal memory encoding phase was further compared in this study to explore any difference in the underlying neural correlates involved in memory formation. Consistent with our hypothesis, individuals with music training demonstrated significantly higher long-range intrahemispheric coherence in the theta frequency band than individuals without music training during the verbal memory encoding phase. Given that intra- and interhemispheric theta coherences at baseline resting state was comparable between two groups, the difference in the long-range intrahemispheric theta coherence during the verbal memory encoding phase was suggestive of an expertise- and task-dependent effect. Moreover, their subsequent memory recall performance was significantly associated with increased long-range left intrahemispheric theta coherence, which is primarily responsible for the learning of verbal material. This finding is in line with a study by Summerfield and Mangels in 2005, in which left long-distance functional coupling was found to be related to successful word encoding and subsequent memory recall [49]. Therefore, this study revealed a neural correlate for enhanced verbal memory performance among individuals with music training, and music training was associated with higher cortical synchronization of the neural networks involved in verbal memory formation. Recent studies have highlighted the importance of the cortical synchronization of long-range theta coherence during memory formation. It has been shown that successful memory encoding is mediated by increased cortical synchronization in the theta frequency band (especially during early word presentation) measured in the intra- and interhemispheric regions [48–50, 52, 70, 82], mostly involving the connection between the frontal and temporoparietal regions. The findings of this study together with those of previous studies are in agreement that these cortical synchronization activities may reflect the central executive integration processes [83, 84] or the interactions between the neocortex and hippocampus [85–87]. Our study provided further robust findings on the association between long-range left intrahemispheric coherence and verbal memory performance in individuals with music training. As suggested in the study by Summerfield and Mangels [49], the involvement of right intrahemispheric theta coherence is more related to the processing of sensory contexts whereas interhemispheric theta coherence is more prominent during the active manipulation of material during a working memory task [44]. The absence of increased right intrahemispheric and interhemispheric theta coherence during the verbal memory encoding phase may be attributed to the requirement of carrying out a simple task in this study during which the participants were requested to remember a list of words that did not involve memorizing of contextual information or manipulation of the material to be learned. In addition, the lack of short-range intrahemispheric coherence found in this study appears to agree with the general trend in the existing literature showing greater involvement of long-range intrahemispheric coherence in the theta frequency band for memory formation, but much less evidence for that of short-range intrahemispheric coherence.

To control for the processing time involved in recalling the verbal material presented in the EEG paradigm, a recognition trial rather than a free recall trial was used, as is common practice in the list learning test to determine the subsequent memory performance of the individuals. The reaction time of each individual during the recognition trial was recorded but showed no significant difference between the MT and NMT groups. Therefore, the better discrimination scores found in the former seem to be independent of the processing time involved during recognition. The discrimination scores in the MT group were not significantly correlated with years of music training. Although a positive association between years of music training and verbal memory performance is found in children [31], our study of adults did not find a similar relationship between years of music training and behavioral or EEG measures. The participants had been engaged in music training for at least one year with the longest duration around 13 years. The non-significant associations between years of music training and behavioral and EEG measures have two implications. First, music training for at least one year may already have induced changes in the cortical synchronization involved in memory formation. This implication is consistent with the longitudinal behavioral study that investigated the effects of music training in children [31]. After one year of music training, children demonstrated better verbal memory compared to their own performance before music training. Those who discontinued the training showed no significant change in verbal memory after one year. Second, a longer duration of music training does not seem to have differential effects on the degree of the cortical synchronization experienced during the memory encoding phase. Our current data suggest that additional years of training may not further enhance verbal memory ability once the cortical synchronization of memory processing has been enhanced after a year of music training. Although a larger sample with a wider range of music training duration may be required to further validate the results, infinite enhancement of memory with increasing years of music training is extremely unlikely. Given the positive effects of music training on brain function, community programs that offer music enrichment opportunities have been launched to help underserved children. Moreover, the neural processing of speech in at-risk children is enhanced [88] and the positive effects of music training on the linguistic abilities of children can be seen within only six months, as demonstrated in a study by Moreno et al. [24]. Therefore, it is worthwhile to conduct further studies on adult individuals to assess both verbal and visual memory along with brain activity by offering one year of music training to look for any change in the behavioral and EEG measures before and after music training.

As short-range theta coherence, particularly over the frontal scalp region, is more usually associated with the neural correlates of an attentional system [83], it is postulated that the insignificant difference in short-range intrahemispheric coherence in the theta frequency band between the MT and NMT groups may suggest similar levels of attentional load allocated by both groups during the verbal memory encoding phase. If this postulation is correct, better memory recall in the MT group is unlikely to be due to better attention. Yet, further studies are warranted to verify this postulation by including the attentional process as a measure. As suggested by Sweeney-Reed et al. [89], greater pre-stimulus theta power in the thalamus was found to be predictive of better memory performance and post-stimulus correlates of successful memory formation. They concluded that these pre-stimulus thalamic theta oscillations reflected brain processes that specifically underpin memory formation. It is implied that increased theta coherence among individuals with music training in this study may also precede the pre-stimulus stage, which warrants further investigation. In addition, it is worthwhile to use source localization analysis in further studies to identify from which specific parts of the brain regions the scalp-measured EEG coherence originates.

Another limitation of this study is that brain activity during recognition was not recorded, but could provide important clues to the differences in the neural correlates of memory performance for successfully recalled versus unrecalled words, and concrete versus abstract words within and between the MT and NMT groups. This study was the first attempt to explore the underlying neural correlates of the verbal memory advantages in the MT group and to associate behavioral memory performance with the cortical synchronization of brain activity during the verbal memory encoding phase. Further investigations to compare brain activity during recognition are necessary to determine whether music training is also associated with the neural correlates of memory retrieval. It should also be noted that all EEG and neuropsychological measures were collected after music training. Thus, the present study cannot draw a causal conclusion about whether music training influences verbal memory performance or if the two groups already differed in EEG patterns, memory functioning, and cognitive capability before the training. Therefore, it is worthwhile to conduct a longitudinal study where pre-training parameters (EEG and cognitive measures) are controlled between individuals with and without music training to investigate the causal relationship between such training and memory enhancement. A future study can also consider adopting a visual memory task that involves a learning trial and a delayed recall trial more comparable to the current verbal memory task to verify whether a modality-specific influence of music training on memory function really exists.

Furthermore, in our sample, only 6 participants were considered to have high musical activity, that is, at least 10 years of playing a musical instrument. The participants also played various types of Chinese or Western musical instruments with the majority being the Western type. As demonstrated in the elderly, different levels of musical activity can preserve cognitive functioning during aging [90]. As the level of participation in musical activity may lead to different degrees of improvement in cognitive functioning, more participants with high musical activity, or a sample with a more even distribution of Western and Chinese musical instruments, should be recruited for comparison in future research to explore the neural correlates that support these differences and the possibility of instrument-dependent influence on the results. Besides, the participant’s average duration of music training and practice per day/ week, which were not collected in the present study, should also be gathered and taken into account. Finally, the possibility of inherited factors (such as genetic pleiotropy) and the gene-environment interaction may have introduced pre-training differences among the individuals with and without music training [91, 92]. As a result, interpretation of this study requires caution and future studies that control for these pre-training differences are warranted.

## Conclusion

In the present study, individuals with music training demonstrated better verbal memory than those without music training during both the learning and the delayed recall trials in the paper-and-pencil tests. Individuals with music training also exhibited greater learning capacity during the learning trials. During the verbal memory encoding phase, they showed an increase in long-range left and right intrahemispheric EEG coherence in the theta frequency band, compared with individuals without music training. More importantly, their event-related left intrahemispheric theta coherence was positively associated with subsequent verbal memory performance as measured by discrimination scores. These findings add to the growing body of music training literature by showing that music training may modulate the cortical synchronization of the neural networks involved in verbal memory formation.
